# Assessing the Impact of a Social Marketing Campaign on Program Outcomes for Users of an Internet-Based Testing Service for Sexually Transmitted and Blood-Borne Infections: Observational Study

**DOI:** 10.2196/11291

**Published:** 2019-01-21

**Authors:** Mark Gilbert, Travis Salway, Devon Haag, Michael Kwag, Joshua Edward, Mark Bondyra, Joseph Cox, Trevor A Hart, Daniel Grace, Troy Grennan, Gina Ogilvie, Jean Shoveller

**Affiliations:** 1 British Columbia Centre for Disease Control Vancouver, BC Canada; 2 School of Population and Public Health University of British Columbia Vancouver, BC Canada; 3 Community Based Research Centre for Gay Men's Health Vancouver, BC Canada; 4 Health Initiative for Men Vancouver, BC Canada; 5 Department of Epidemiology, Biostatistics and Occupational Health Faculty of Medicine McGill University Montreal, QC Canada; 6 Department of Psychology Ryerson University Toronto, ON Canada; 7 Dalla Lana School of Public Health University of Toronto Toronto, ON Canada; 8 Division of Infectious Diseases Faculty of Medicine University of British Columbia Vancouver, BC Canada

**Keywords:** advertisements, diagnostic tests, health promotion, internet, men who have sex with men, social marketing

## Abstract

**Background:**

While social marketing (SM) campaigns can be effective in increasing testing for sexually transmitted and blood-borne infections (STBBIs), they are seldom rigorously evaluated and often rely on process measures (eg, Web-based ad click-throughs). With Web-based campaigns for internet-based health services, there is a potential to connect campaign process measures to program outcomes, permitting the assessment of venue-specific yield based on health outcomes (eg, click-throughs per test).

**Objective:**

This study aims to evaluate the impact of an SM campaign by the promotional venue on use and diagnostic test results of the internet-based STBBI testing service GetCheckedOnline.com (GCO).

**Methods:**

Through GCO, clients create an account using an access code, complete a risk assessment, print a lab form, submit specimens at a lab, and get results online or by phone. From April to August 2015, a campaign promoted GCO to gay, bisexual, and other men who have sex with men in Vancouver, Canada. The campaign highlighted GCO’s convenience in 3 types of promotional venues—location advertisements in print or video displayed in gay venues or events, ads on a queer news website, and ads on geosocial websites and apps. Where feasible, individuals were tracked from campaign exposures to account creation and testing using venue-specific GCO access codes. In addition, Web-based ads were linked to alternate versions of the campaign website, which used URLs with embedded access codes to connect ad exposure to account creation. Furthermore, we examined the number of individuals creating GCO accounts, number tested, and cost per account created and test for each venue type.

**Results:**

Over 6 months, 177 people created a GCO account because of the campaign, where 22.0% (39/177) of these completed testing; the overall cost was Can $118 per account created and Can $533 per test. Ads on geosocial websites and apps accounted for 46.9% (83/177) of all accounts; ads on the news website had the lowest testing rate and highest cost per test. We observed variation between different geosocial websites and apps with some ads having high click-through rates yet low GCO account creation rates, and vice versa.

**Conclusions:**

Developing mechanisms to track individuals from Web-based exposure to SM campaigns to outcomes of internet-based health services permits greater evaluation of the yield and cost-effectiveness of different promotional efforts. Web-based ads with high click-through rates may not have a high conversion to service use, the ultimate outcome of SM campaigns.

## Introduction

Social marketing (SM) campaigns promoting testing for sexually transmitted and blood-borne infections (STBBIs) can effectively increase the uptake of testing [1,2]. However, SM testing campaigns are rarely evaluated rigorously owing to pressures of real-world implementation (eg, evaluation budget and difficulty determining campaign-specific effects in an exposed population) [1,3]. Web-based elements of SM campaigns are often evaluated through monitoring the number of views (impressions) of Web-based ads and comparing the proportions of individuals clicking through to visit (click-through rate) and use a website or service (conversion rate) [4,5]; this information is used to identify promotional venues with higher yield, allowing redirection of efforts to optimize campaign reach and inform future campaigns [1].

More robust evaluations of SM campaigns are possible for campaigns promoting internet-based health services, where users are tracked through service progression. If designed appropriately, campaign evaluations can follow individuals from their initial campaign ad view through to their program outcomes, permitting an assessment of yield of different venues based on actual health outcomes. This paper aims to describe the results of using such a design to evaluate the impact of an SM campaign on increasing the uptake of GetCheckedOnline.com (GCO) [6], an internet-based STBBI testing service in British Columbia (BC), Canada.

## Methods

### GetCheckedOnline

GCO is an internet-based testing service for STBBIs developed by the BC Centre for Disease Control (BCCDC), with a goal of overcoming existing testing barriers among populations with high rates of infection. We have previously published a full description of the GCO program [7]. In brief, users go through the following 5 steps to test through GCO: (1) create an account; (2) complete a risk assessment; (3) print a laboratory requisition form; (4) provide specimens in-person at a private lab (with testing for HIV, syphilis, hepatitis C, chlamydia, and gonorrhea); and (5) receive results online if negative, or by phone if positive or indeterminate. GCO accounts are created by entering an access code on the home page unique to a specific promotion strategy or venue. In addition, individuals can be invited to use GCO by emails with a link to the account creation page.

GCO was launched in 2014, initially targeting gay, bisexual, and other men who have sex with men (GBMSM) in the Vancouver region. Most GBMSM in BC regularly test, with 64.57% (1179/1826) and 62.28% (999/1604) reporting sexually transmitted infection and HIV testing, respectively, in the past year [8]. However, many GBMSM report delaying testing owing to barriers, including privacy concerns or inability to access clinics [9]. In formative research, GBMSM found GCO acceptable with high intention to use, particularly among men facing testing barriers, perceiving benefits, including greater privacy, convenience, control over testing, and not needing to see a health care provider [9,10].

### Campaign Development

**Figure 1:**
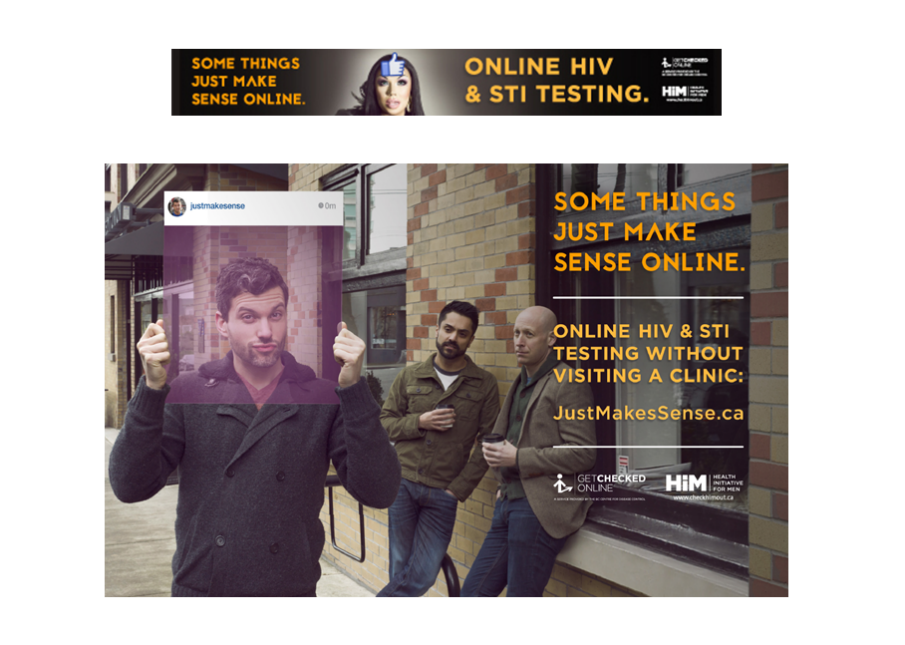
Examples of promotional campaign materials used for an internet-based testing service for sexually transmitted and blood-borne infections (GetCheckedOnline.com), including online banner advertisements (top), and in print (bottom).

The SM campaign aimed to increase the awareness and uptake of GCO among GBMSM. The BCCDC partnered with the Health Initiative for Men, a community-based gay men’s health organization, which led the development and implementation of the campaign in consultation with an advisory committee of GBMSM, sexual health nurses, and a small convenience sample of Health Initiative for Men clinic clients. The campaign focused on promoting the convenience of GCO, aiming to reach GBMSM avoiding or delaying testing because of access-related barriers (eg, wait-times for appointments). The campaign concept (Figure 1) was “Some things just make sense online,” designed to use humor based on popular social media sites to motivate viewers to visit the JustMakesSense (JMS) campaign website [11] which emphasized the convenience and confidentiality of the service. Campaign materials included a website, videos, Web-based ads, and print media.

### Tracking Program Outcomes by the Promotional Venue

**Figure 2:**
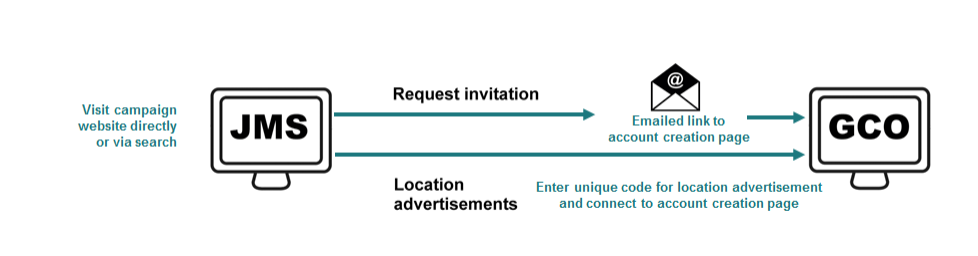
The description of routes to account creation on GetCheckedOnline.com (GCO) during the JustMakesSense (JMS) campaign; visitors to the JMS main website.

**Figure 3:**
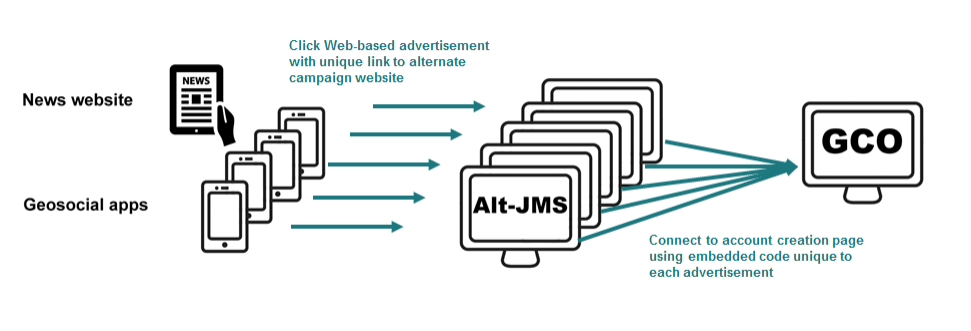
The description of routes to account creation on GetCheckedOnline.com (GCO) during the JustMakesSense (JMS) campaign; visitors from online promotional venues to alternate JMS sites (Alt-JMS).

The campaign ran from April to August 2015. We used 3 venue types for promotion, each having a unique route to account creation, permitting us to track testing outcomes (Figures 2 and 3). *Location ads* included the JMS website address and an access code unique to each location (ie, gay bars and clubs; sex on premises venues; community spaces; businesses; and a lesbian, gay, bisexual, and transgender film festival). Codes were short, easy-to-remember phrases, such as “TestNow” or “TestOnline,” displayed on videos, posters, or postcards (the latter could be taken home by individuals). On the JMS website, visitors entered an access code and proceeded to the account creation page on GCO. Visitors without a code could request an email invitation; visitors were not asked whether or where they had seen the campaign (Figure 2). In addition, we used 2 types of Web-based promotional venues—advertising in a *lesbian, gay, bisexual and transgender news website*, and advertising on *geosocial websites and apps* used by GBMSM to find sex partners (Grindr, Jack’d, Manhunt, Squirt, and Scruff). To track testing outcomes for each Web-based venue, each post or ad contained a link to a unique, alternate copy of the JMS website. From each alternate site, visitors proceeded to the GCO account creation page by clicking a link containing an embedded access code unique to each Web-based venue, which could then be associated with each account created.

### Data Analysis

Our primary outcome was the number of individuals creating GCO accounts by promotional venue type; secondary outcomes included the number of individuals tested, and costs per account created and individual tested. We collected available data from website or app vendors on impressions and click-through rates and extracted GCO program data. For each type of promotional venue, we calculated the number of GCO accounts created and proportion completing testing. For Web-based venues, we described the number of campaign impressions and click- throughs to alternate JMS campaign websites. Furthermore, we described the number of visitors creating accounts through requesting an invitation on the campaign website and their testing outcomes.

### Ethics

Our analysis was conducted under a program evaluation mandate using data routinely collected by BCCDC or through contracts with Web-based ad vendors. The use of individual-level GCO program data is permitted for evaluation under the terms of service agreed to by all GCO users.

## Results

Overall, 177 individuals created a GCO account because of the campaign, and 22.0% (39/177) of these completed testing; all results were negative (Table 1). The highest number of accounts was from individuals viewing campaign images on geosocial apps (83/177, 46.9%) followed by individuals requesting an invitation from the campaign website (52/177, 29.4%), location ads (21/177, 11.9%), and a news website (20/177, 11.3%). The completion of testing showed little variation across venues, except the news website (1/20, 5%). We spent Can $20,801 on promotion; the average cost was Can $118 per account created and Can $533 per test (Table 2). The costliest approach per account created was geosocial apps (Can $211), followed by Web-based news (Can $105) and location ads (Can $53). Web-based news had the highest cost per test (Can $2104). Over 19 million impressions of the JMS campaign occurred through geosocial apps, with the highest click-through rate on Grindr (0.7%). The highest numbers of accounts were created from ads on Manhunt and Squirt, resulting in low costs per account created (Can $83 and Can $213, respectively).

**Table 1:** Outcomes by the promotional venue.

Promotional venue		Number exposed to campaign or Number of impressions	Number visited JMS websites (% of click-through)	Number created account (% of visited JustMakesSense site)	Number completed testing (% of accounts created)
Requested invitation		N/A^a^	N/A	52^b^	14 (26.92)
Location ads		N/A	N/A	21^c^	5 (23.80)
News website		195,120	260 (0.13)	20 (7.69)	1 (5.00)
**Geosocial apps (all)**		19,232,363	41,227 (0.21)	83 (0.20)	19 (22.89)
	Grindr	3,443,423	24,975 (0.72)	5 (0.02)	2 (40.00)
	Jack’d	366,744	655 (0.18)	1 (0.15)	0 (0.00)
	Manhunt	299,284	547 (0.18)	33 (6.03)	11 (33.33)
	Squirt	1,355,044	1,822 (0.13)	37 (2.03)	6 (16.21)
	Scruff	13,767,868	13,228 (0.10)	7 (0.05)	0 (0.00)
Total		19,427,483	41,487 (0.21)	177 (0.30)	39 (22.03)

^a^N/A: not applicable.

^b^These individuals were not asked whether they were exposed to the campaign when they requested an invitation; hence we were unable to estimate the denominator (ie, the number exposed to the campaign) and report % values.

^c^We were unable to estimate the denominator (ie, the number exposed to location ads) and report % values for this.

**Table 2:** Costs by the promotional venue.

Promotional venue			Cost of promotion (Can $)		Cost per account (Can $)		Cost per test (Can $)
Location ads			1115		53		223
Web-based news			2104		105		2104
**Geosocial apps (all)**			17,582		211		925
	Grindr	4270		854		2135	
	Jack’d	3200		3200		N/A^a^	
	Manhunt	7040		213		640	
	Squirt	3072		83		512	
	Scruff	0		0		N/A	
Total			20,801		118		533

^a^N/A: not applicable.

## Discussion

While commonly applied in e-commerce (eg, linking ad exposures to Web-based purchases), this study demonstrated the value of using this evaluation method to understand the effects of campaign ads (in a range of promotional venues) on internet-based health services. For example, just under half of all GCO accounts were created as a result of ads on geosocial apps, where we spent the bulk of our promotional budget. While Grindr had the highest click-through rate and would, therefore, typically be considered a successful promotional venue, Grindr had the lowest proportion of GCO accounts created and a higher cost per account and per test. Conversely, Manhunt and Squirt had lower click-through rates but had higher yield in terms of GCO program outcomes. In addition, we observed the highest account creation rate among individuals exposed to our Web-based news advertisement, although a much lower proportion proceeded to test. These differences in outcomes might be explained by several factors, including the characteristics of GBMSM on these different websites and apps, such as differences in response to the JMS campaign (influencing click-through rates); demographic factors (eg, age and ethnicity); and behavioral risk or testing barriers (influencing account creation and testing rates)—all aspects worthy of further study [12,13]. Furthermore, our findings demonstrate that the promotion in physical venues is important and cost-effective, as location ads had the lowest cost per account of all venues. However, we were unable to account for view through conversion, where GBMSM seeing campaign ads may have later requested a GCO invitation on the campaign website (29% of all accounts created).

We did not observe a large uptake in testing as a result of the JMS campaign. The 39 individuals testing through GCO may be “early adopters” of this intervention with the ongoing diffusion of this innovation through GBMSM networks [14]. A shift in the message may also be needed. The feedback from GBMSM and providers following the campaign suggested convenience may not be the best selling point, given the relative availability of STBBI testing services for GBMSM in the Vancouver area (Edwards J, personal communication, November 2016); this may explain why only 1 in 5 men creating accounts tested through GCO, a measure associated with motivation to get tested in our prior evaluations [15].

In conclusion, this study demonstrates the value of developing mechanisms for tracking individuals from their Web-based exposure to SM campaign ads about an internet-based health service to their program outcomes. In addition, this study reveals that Web-based venues with high click-through rates may not always have a high conversion to service use, which is ultimately the desired outcome of SM campaigns. We are continuing to use venue-specific access codes to evaluate promotional efforts as GCO expands to other communities across BC.

## Abbreviations

BC: British Columbia
BCCDC: British Columbia Centre for Disease Control
GBMSM: gay, bisexual and other men who have sex with men
GCO: GetCheckedOnline.com
JMS: JustMakeSense
SM: Social marketing
STBBI: sexually transmitted and blood-borne infection
